# Natural Resonance Regulation of Ce_x_Ba_1-x_Co_2_Fe_16_O_27_ Hexaferrite via In Situ Doping Strategy for Efficient Microwave Absorption

**DOI:** 10.3390/nano16211356

**Published:** 2026-10-25

**Authors:** Mian Sun, Yan Sun, Linghua Tan, Leichong Wang

**Affiliations:** 1 School of Chemistry and Chemical Engineering, Nanjing University of Science and Technology, Nanjing 210094, China; sunm@njust.edu.cn (M.S.); sun7512388@163.com (Y.S.); 2 School of Safety Science and Engineering, Nanjing University of Science and Technology, Nanjing 210094, China; 3 Advanced Engineering Technology Research Institute of Zhongshan City, Wuhan University of Technology, Zhongshan 528437, China; lc.w@foxmail.com; 4 Precision Concrete New Material Technology (Guangdong) Co., Ltd., Zhongshan 528400, China

**Keywords:** in situ doping strategy, hexaferrite, natural resonance frequency, rare earth, microwave absorption

## Abstract

Recently, hexagonal ferrites (hexaferrites) have attracted increasing attention for high-performance microwave absorption owing to their favorable magnetic and dielectric properties. However, their natural resonance in the GHz region limits their application in narrow-band high-frequency absorbers. Herein, Ce^3+^-doped BaCo_2_Fe_16_O_27_(CBCF) hexagonal ferrite sheets with varying Ce concentrations were successfully synthesized via a sol–gel method combined with high-temperature calcination. With increasing Ce^3+^ content, the diffraction peaks shifted towards higher angles, and the magnetic parameters of the W-type hexaferrite were effectively improved. The saturation magnetization increased from 75.66 to 83.73 emu/g. Notably, the Ce_0.075_Ba_0.925_Co_2_Fe_16_O_27_(C_0.075_BCF) sample exhibited excellent broadband absorption performance, achieving a minimum reflection loss (RL) of −18.40 dB at 14.92 GHz and an effective absorption bandwidth of 5.52 GHz. The microwave absorption performance was effectively controlled by tuning the natural resonance frequency of the W-type hexaferrite via Ce doping. This study provides a new strategy for designing broadband microwave absorbers by modulating the natural resonance frequency.

## 1. Introduction

With the development of the electromagnetic industry, electromagnetic wave (EMW) pollution in the GHz range has been a major concern in the fields of integrated microelectronic devices, information warfare and human health [1,2]. Thus, researchers have been developing different categories of electromagnetic-wave-absorbing (EWA) materials such as hexaferrite, graphene, conducting polymer and transition metal sulfide [3,4,5,6,7]. Recently, significant progress has been made in developing advanced microwave absorbers and electromagnetic interference (EMI) shielding materials to achieve broader bandwidths and stronger attenuation capabilities [8,9]. Hexagonal ferrite, with high magnetic permeability, strong anisotropy, stable chemical properties and low price, is considered a potential high-frequency absorber [10,11].

M-type, W-type, Y-type and Z-type hexaferrites have been widely studied for microwave absorption applications [12]. M-type hexaferrites (e.g., BaFe_12_O_19_ and SrFe_12_O_19_) are the most widely studied due to their low cost and high Curie temperature [13]. W-type hexaferrites (e.g., BaCo_2_Fe_16_O_27_) exhibit superior magnetic properties owing to their higher saturation magnetization, making them promising candidates for high-frequency absorbers [14]. W-type hexaferrites typically exhibit planar or cone magnetic anisotropy, and their anisotropy symmetry is primarily determined by Co^2+^ ions at octahedral sites, which governs the soft magnetic properties and high-frequency resonance behavior. The substitution of Ba^2+^ by Ce^3+^ can not only modify the lattice parameters but also influence the magnetocrystalline anisotropy field and natural resonance frequency by altering the chemical states of Co and Fe. However, the intrinsic natural resonance frequency of W-type hexaferrites typically exceeds 17 GHz, limiting their application in the 2–18 GHz range [15,16]. This constraint necessitates effective strategies to shift the natural resonance frequency toward lower GHz bands [17].

The latest research indicates that the microwave absorption mechanisms of magnetic materials include domain wall resonance, hysteresis, eddy current effect and natural resonance, wherein natural resonance dominates mainly the loss in the frequency of GHz [18,19]. At the natural resonance frequency, the imaginary permeability which represents the ability to consume magnetic field energy reaches the maximum [20]. Recent studies have shown that the natural resonance frequency of magnetic material is mainly affected by coercivity and magnetic anisotropy [21]. Gorbachev et al. established a connection between coercivity and natural resonance frequency of M-type hexaferrite [13]. The research results indicate that the change in natural resonance frequency is consistent with coercivity. Cheon et al. studied the connection between magnetic anisotropy and natural resonance frequency [14]. Now, adjusting the natural resonance frequency of magnetic material is essential to achieve the enhancement of permeability in a specific frequency band [22].

Two primary strategies have been explored to modulate the natural resonance frequency of hexaferrites [23]. The first approach involves morphological engineering, such as flattening or rod-like structuring, which introduces shape anisotropy and effectively shifts the resonance frequency [24]. For instance, Xu et al. synthesized CoFe_2_O_4_ ferrite nanorods with the natural resonance frequency shifted to 6.5 GHz [25]. The second approach is compositional engineering via element substitution (doping), which alters the magnetocrystalline anisotropy field and coercivity [26]. Doping with rare-earth ions such as Dy^3+^, Nd^3+^ and Pr^3+^ has been shown to reduce the anisotropy field and generate multiple resonance modes, thereby broadening the absorption bandwidth [27]. In situ doping is an effective approach, offering the advantage of achieving homogeneous heteroatom distribution, enabling more precise property modulation [28].

Despite these advances, several challenges remain unresolved. The relationship between Ce^3+^ doping concentration and the evolution of unit cell parameters, coercivity and the anisotropy field in W-type hexaferrites has not been systematically investigated [29]. Furthermore, the mechanism by which Ce^3+^ substitution induces dual natural resonance peaks and broadens the effective absorption bandwidth remains unclear [23]. Finally, the optimal doping level required to achieve both impedance matching and maximum attenuation has yet to be established [30]. For these reasons, this study aims to prepare CBCF via an in situ doping strategy and elucidate the effects of Ce^3+^ concentration on its structural, magnetic and microwave absorption properties.

Herein, the hexaferrites (CBCF) are prepared by in situ doping strategy. The design concept is to control substitution into the crystal lattice and adjust the microstructure and composition by controlling the Ce^3+^ doping concentration, aiming to adjust the natural resonance frequency. In this context, the effect of resonance frequency on the microwave absorption properties for CBCF is further elucidated. The results show that Ce^3+^ doping induces a reduction in the crystal cell parameters, enhances the super-exchange interaction between Fe-O-Fe, and increases the saturation magnetization strength. Simultaneously, the decrease in the amount of Fe^3+^ and the generation of Fe^2+^ effectively reduces the natural resonance frequency of CBCF and strengthens the electromagnetic characteristics. The C_0.075_BCF exhibited excellent broadband absorption performance, with an RL of −18.40 dB at 14.92 GHz and an effective absorption bandwidth of 5.52 GHz. This research provides a new perspective on the preparation of high-performance broadband microwave absorbers based on the tuning of the natural resonance frequency by controlling the Ce doping.

## 2. Materials and Methods

### 2.1. Materials

Barium nitrate (Ba(NO_3_)_2_), cerium nitrate hexahydrate (Ce(NO_3_)_3_·6H_2_O), iron nitrate nonahydrate (Fe(NO_3_)_3_·9H_2_O), cobalt nitrate hexahydrate (Co(NO_3_)_2_·6H_2_O), citric acid monohydrate (C_6_H_8_O_7_·H_2_O) and ammonia (NH_3_·H_2_O) were purchased from Shanghai Macklin Biochemical Co. Ltd. (Shanghai, China).

### 2.2. Synthesis of Ce_x_Ba_1-x_Co_2_Fe_16_O_27_

**Figure 1:**
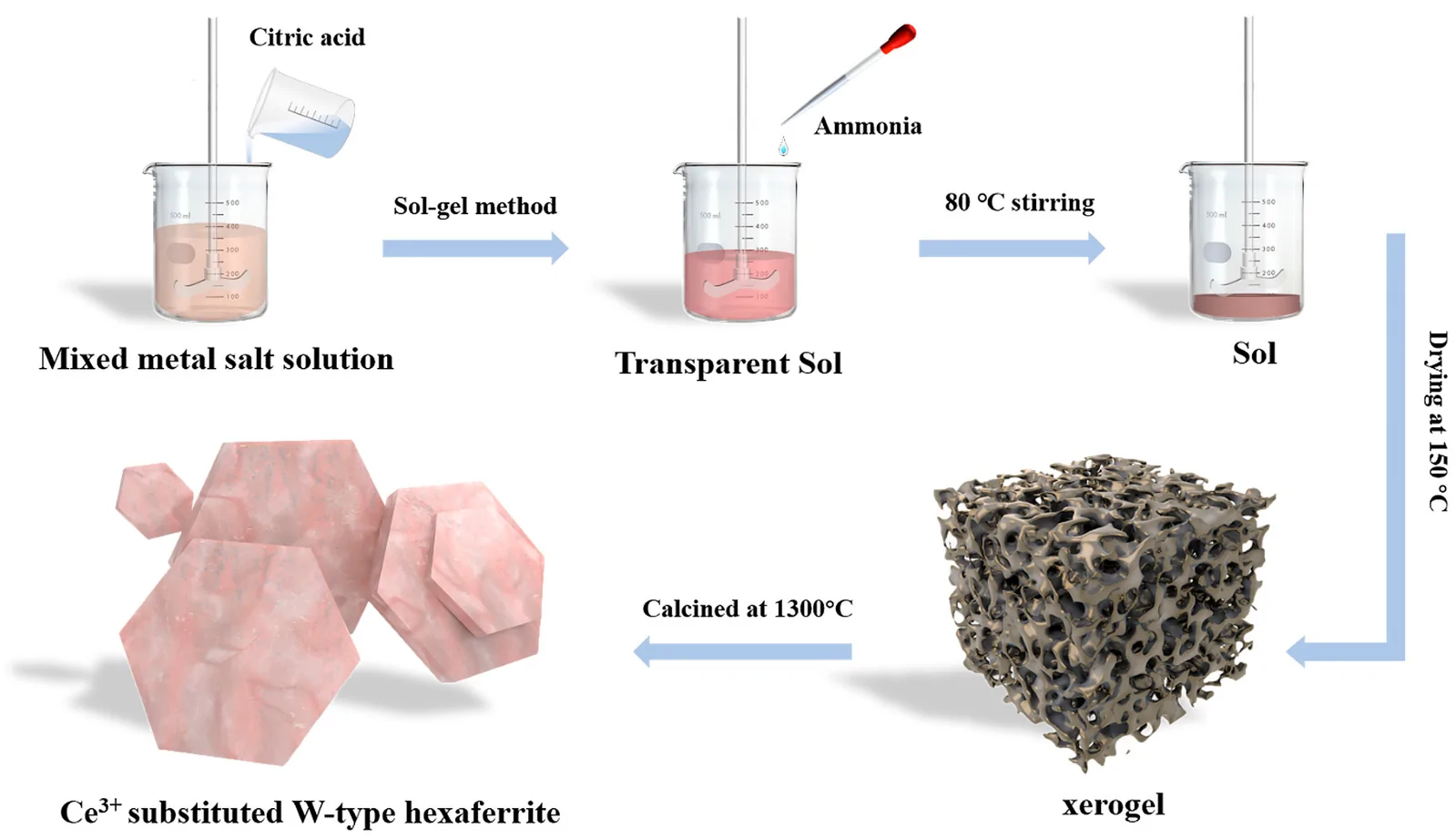
The synthetic route of Ce^3+^ doped W-type hexaferrite. The straight blue arrows indicate the direction of the experimental steps.

As shown in Figure 1, C_x_B_1-x_CF (x = 0, 0.025, 0.050, 0.075, 0.100, 0.125) were prepared by the sol–gel method. First, cerium nitrate, barium nitrate, iron nitrate, and cobalt nitrate were dissolved in deionized water and named metal salt solution A. The molar ratio of Ce^3+^:Ba^2+^:Co^2+^:Fe^3+^ was designed to be x:1-x:2:15.3 (x = 0, 0.025, 0.050, 0.075, 0.100, 0.125). Second, citric acid was added to deionized water, and the result was slowly added to solution A. The molar ratio of citric acid to metal ions was 1.2:1. The mixed solution was stirred for 30 min. Ammonia was added into the mixed solution to adjust the pH to 6–7, forming solution B. Solution B was heated at 80 °C until it formed a green sol. The green sol was transferred to the dried gel at 150 °C for 12 h. Finally, the dried gel was calcined to form Ce^3+^-doped W-type hexaferrite at 1300 °C for 3 h. In this paper, W-type hexaferrite with a series of doping levels was named BCF, C_0.025_BCF, C_0.050_BCF, C_0.075_BCF, C_0.100_BCF, C_0.125_BCF.

### 2.3. Sample Characterization

(1) X-ray Diffraction (XRD)

The phase composition of the CBCF materials was analyzed using an AXS D8 Advance X-ray diffractometer (Bruker, Karlsruhe, Germany) with Cu Kα radiation. The scanning range was 10–80°, the scanning rate was 2°/min, and the step size was 0.02°. GSAS-II software (version 5438) was used for Rietveld refinement of the CBCF materials with different doping amounts.

(2) Scanning Electron Microscopy (SEM)

The CBCF materials were placed on conductive tape and sputter-coated with gold prior to morphological characterization. The surface morphology was analyzed using a scanning electron microscope (Merlin Compact, Carl Zeiss, Oberkochen, Germany) with a magnification range of 500 nm–20 μm.

(3) X-ray Photoelectron Spectroscopy (XPS)

The surface chemical states of the Ce_0.075_BCF material were analyzed using an ESCA-2000 X-ray photoelectron spectrometer (Thermo Fisher Scientific, Waltham, MA, USA) equipped with monochromatic Al Kα radiation (1486.6 eV). All measurements were performed in hybrid mode with a take-off angle of 90° and a spot size of 400 μm.

(4) Vibrating Sample Magnetometer (VSM)

The static magnetic properties of the samples were characterized using a Lakeshore 7400-S VSM (Lake Shore Cryotronics, Westerville, OH, USA). The sample mass was 15 mg, and the magnetic field range was −20 kOe to 20 kOe.

(5) Vector Network Analyzer (VNA)

The microwave absorption properties of the samples were analyzed using an Agilent N5230C vector network analyzer (Agilent Technologies, Santa Clara, CA, USA). The coaxial method was employed in this experiment, and the testing range was 2–18 GHz. The powder samples were uniformly mixed with paraffin wax and pressed into toroidal-shaped specimens. Prior to measurement, a full two-port TRL calibration was performed to establish the measurement reference plane at the sample surfaces. The S-parameters were recorded, and the complex permittivity and permeability were extracted using the NRW algorithm.

The reflection loss (R_L_) which was used to indicate the microwave absorbing properties was calculated by the Equation (1):

*R*_L_ = 20log|(Z_in_ − Z_0_)/(Z_in_ + Z_0_)| (1)

where Z_in_ is the input impedance of the absorber and Z_0_ is the impedance of free space. Z_in_ can be obtained from following equation:

(2) $$Z_{\mathrm{in}}{\;=\;Z}_{0}{{(\mu }_{r}{/\varepsilon }_{r})}^{1/2}\tan h[j\left(2\pi fd/c\right)\left(\mu_{r}\varepsilon_{r}{)}^{1/2}\right]$$

where c is the velocity of electromagnetic waves in free space and d is the absorber thickness.

## 3. Results

### 3.1. Morphological and Structural Characteristics

**Figure 2:**
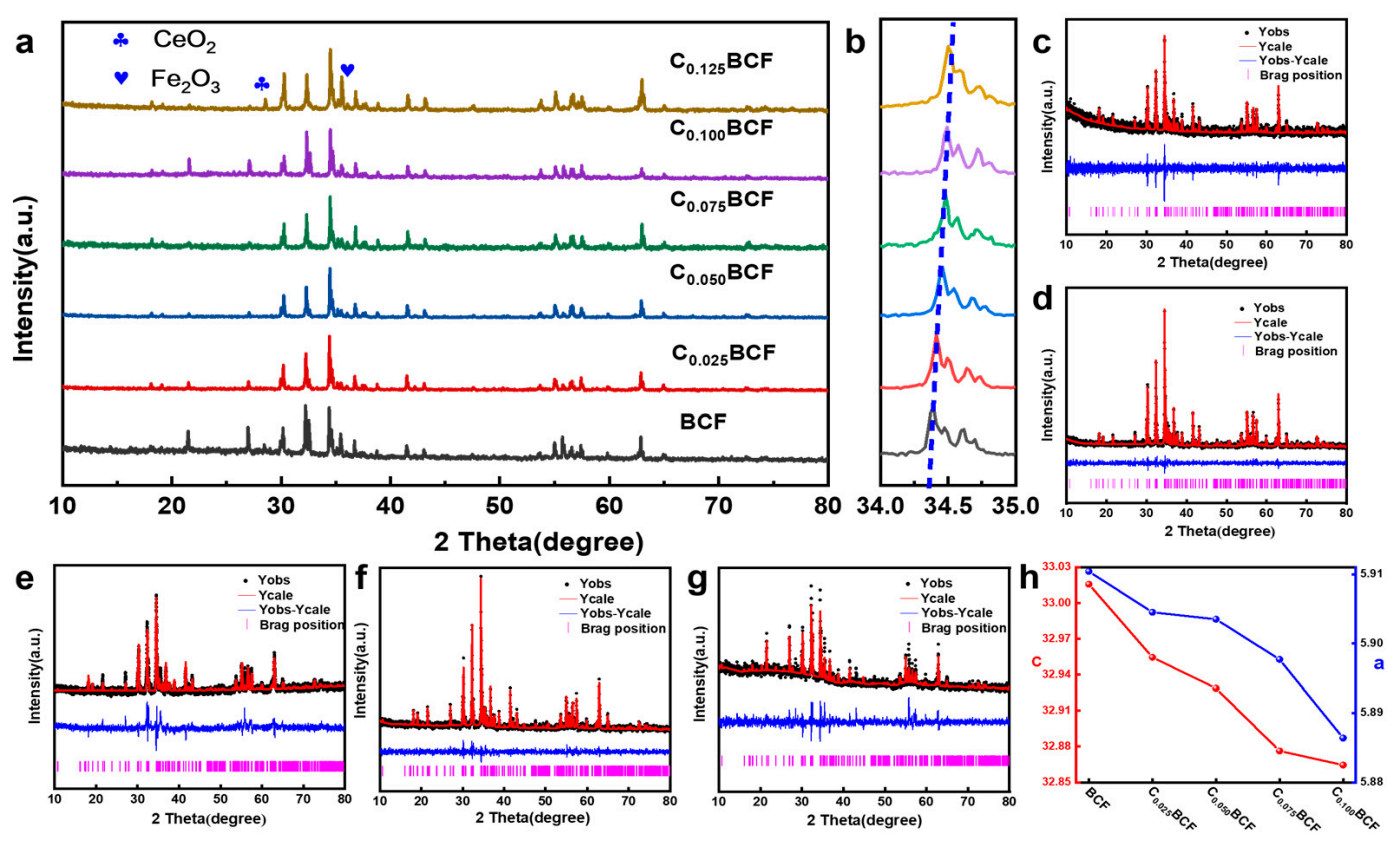
The Structure of CBCF materials. (**a**) XRD patterns; (**b**) enlarged XRD patterns; (**c**–**g**) The Rietveld refined patterns; (**h**) lattice parameters (**a**,**c**) of CBCF. In (**a**), the blue diamonds and dark blue inverted triangles indicate the impurity phases of CeO_2_ and Fe_2_O_3_, respectively. In (**c**–**g**), the black dots, red lines, blue lines, and pink vertical bars represent the observed data (Yobs), calculated data (Ycalc), difference between observed and calculated data (Yobs-Ycalc), and Bragg positions, respectively.

Figure 2a displays the X-ray patterns of C_x_BCF (x= 0.000, 0.025, 0.050, 0.075,0.100, 0.125) across the 2θ range of 10–80°. As shown in Figure 2a, the diffraction peaks at 30.28°, 32.38°, 34.43°, and 62.98° correspond to the (110), (1010), (116), and (220) crystal planes of W-type hexaferrite, which is consistent with the standard card PDF#78-1536 [31]. This indicates that the CBCF belongs to the P63/mmc space group [32]. However, the extra diffraction peaks at 29.32°,35.82° of CeO_2_ and Fe_2_O_3_ were observed in C0.125BCF materials [33,34,35,36]. The extra peaks occur because the Ce ions reached the dissolution limit in W-type hexaferrite [33,37]. Figure 2b shows the enlarged XRD patterns of CBCF samples in the 2θ range of 34–35°. The peaks of CBCF tend to shift towards a higher angle as Ce content increases, which may be attributed to differences in the ionic radius between Ce and Ba [36,38]. The ionic radius of Ce^3+^ is 1.04 Å, which is smaller than that of Ba^2+^, and causes the decrease in the lattice parameter [38,39].

The Rietveld refined patterns of the CBCF materials are shown in Figure 2c–g. The values of lattice parameters (a,c) and cell volume (Vcell) are shown in Table 1. Considerably low values of the fitting refinement parameters suggested that the experimental and fitted graphs of the CBCF material can overlap well [40,41], as shown in Figure 2c–g. Figure 2h shows that as Ce ion content increases, the lattice parameters a and c decrease significantly, which leads to the increase in X-ray diffraction peaks (Figure 2b) [39]. This suggests that the Ce^3+^ ions have successfully substituted for the Ba^2+^ in the BaCo_2_Fe_16_O_27_ lattice [41].

**Figure 3:**
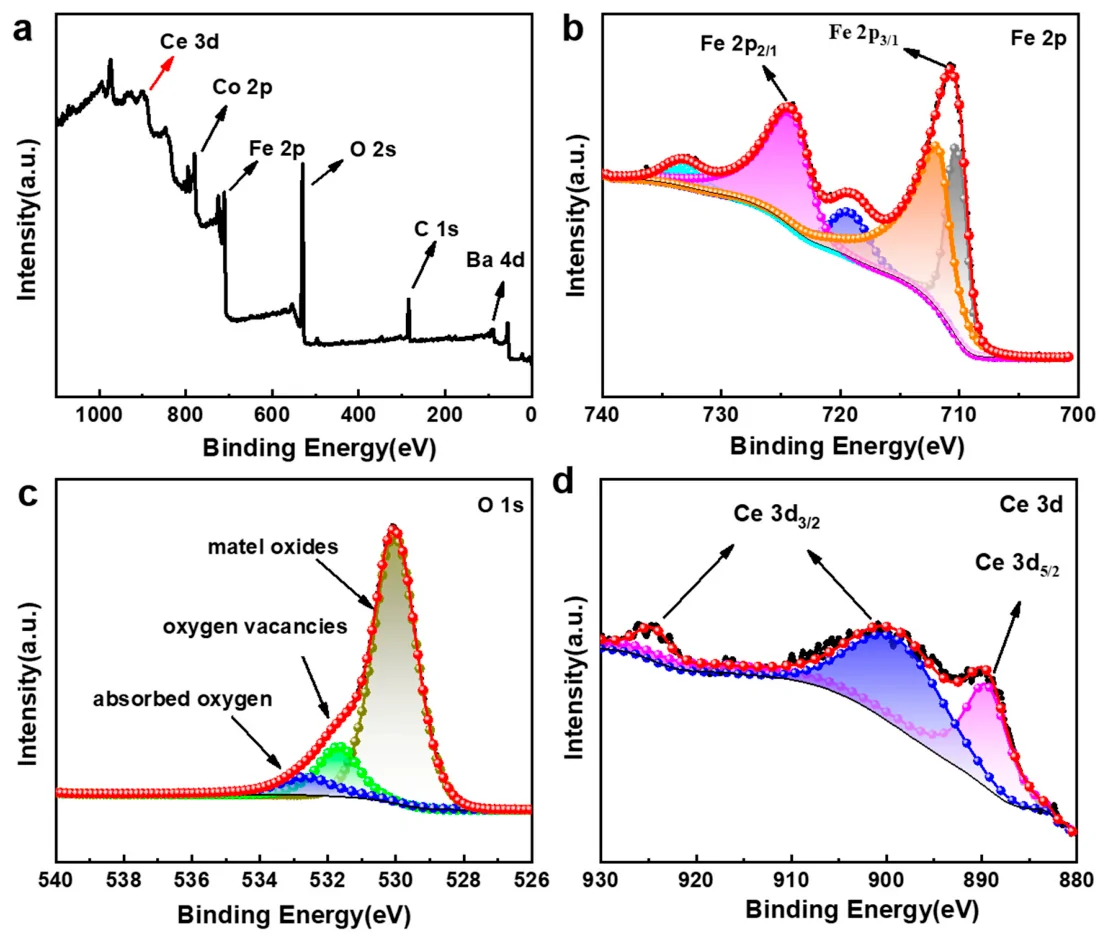
XPS spectra of (**a**) the survey spectrum, and high-resolution spectra of (**b**) Fe 2p, (**c**) O 1s, and (**d**) Ce 3d for the C_0.075_BCF sample. In (**c**), the colored fitted peaks represent metal oxides (lattice oxygen), oxygen vacancies, and adsorbed oxygen, respectively. In (**d**), the fitted peaks correspond to the Ce 3d_3/2_ and Ce 3d_5/2_ spin-orbit components.

The elemental valence states of the C_0.075_BCF were investigated by XPS. Figure 3a depicts the scan survey spectrum of the CBCF materials, which showed evidently characteristic peaks of Ba, Co, Fe, O. The element of Ce displays a weak peak because of the low content [42]. As shown in Figure 3b, the high-resolution Fe 2p spectrum exhibits two main peaks located at approximately 711 eV and 724.5 eV, which are assigned to Fe 2p_3/2_ and Fe 2p_1/2_, respectively, due to the spin-orbit coupling. Furthermore, the distinct satellite peaks observed at around 716.26 eV and 732.97 eV are characteristic features corresponding to the Fe^2+^ and Fe^3+^ states, respectively. The presence of these specific satellite peaks clearly confirms the coexistence of both Fe^2+^ and Fe^3+^ in the sample [43]. This coexistence is attributed to the charge compensation mechanism induced by the heterovalent substitution of Ba^2+^ by Ce^3+^ [44]. Figure 3c shows the narrow spectrum of O 1s. The peaks in the range of 526–540 eV indicate that three states of O existed: lattice oxygen (Fe-O), oxygen vacancies (O_2_^2−^/O^−^), and adsorbed oxygen (OH^−^/O_2_) [42]. From Figure 3d, the peaks at 898 eV and 887 eV in the Ce 3d spectrum depict the corresponding peaks of Ce 3d_3/2_ and Ce 3d_5/2_. This indicates that the Ce element in the C_0.075_BCF material exists in its trivalent state [34,45]. These results prove that Ce ions successfully substituted for the Ba sites of W-type hexaferrite.

Based on the XPS analysis, the substitution of Ba^2+^ by Ce^3+^ induces a charge imbalance, which is compensated for by the partial reduction of Fe^3+^ to Fe^2+^, as evidenced by the satellite peaks in the Fe 2p spectrum. This change in the chemical state of Fe ions alters the super-exchange interaction between Fe^3+^-O-Fe^3+^ and affects the local coordination environment of Co^2+^ at octahedral sites. Since the planar magnetic anisotropy of W-type hexaferrite mainly originates from Co^2+^ ions occupying octahedral sites, the Ce-induced lattice contraction and Fe valence variation are expected to reduce the magnetocrystalline anisotropy field (H_a_), which is also supported by the calculated H_a_ values in Table 2.

**Figure 4:**
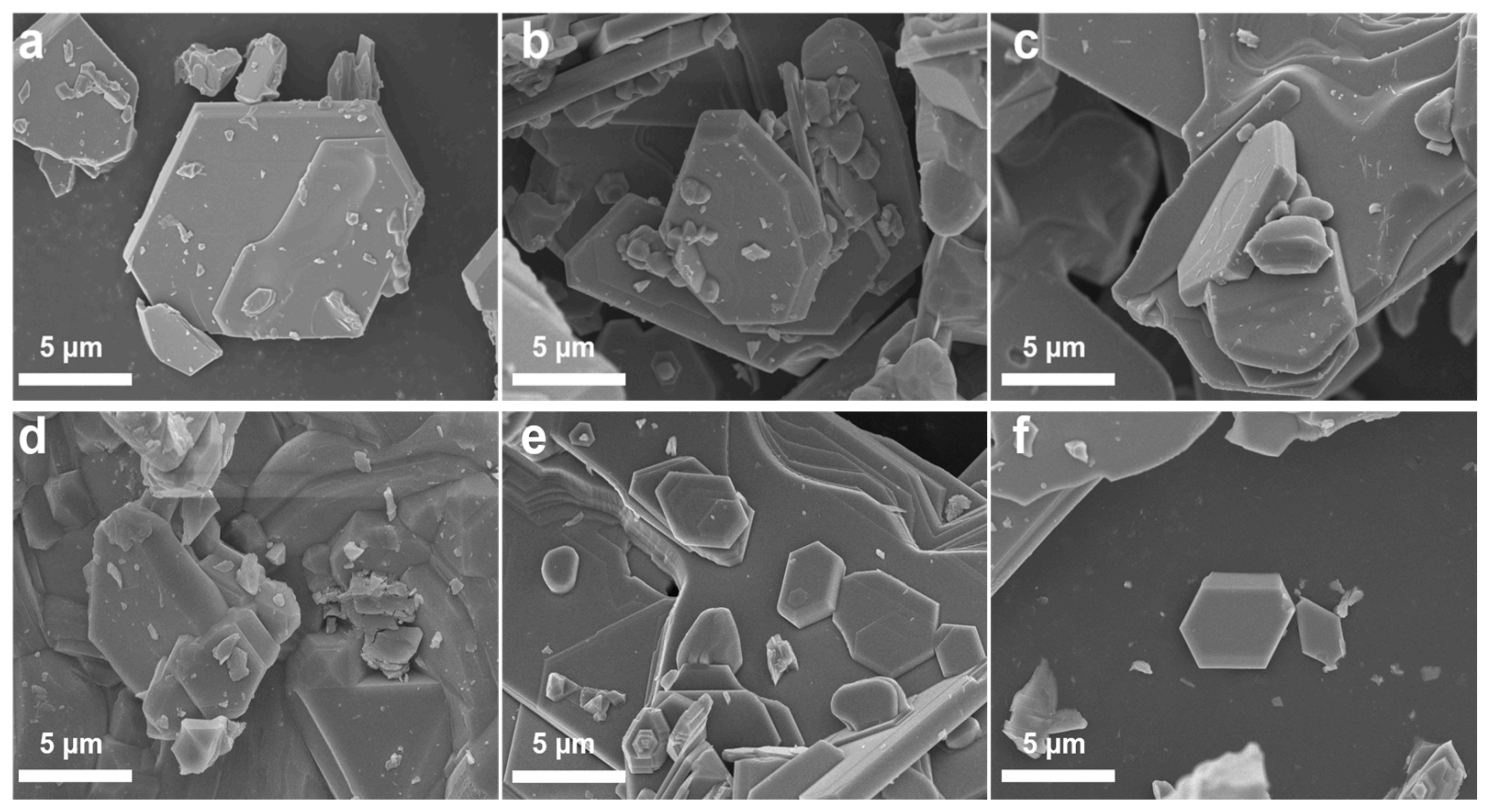
FESEM of (**a**) pure BCF; (**b**) C0.025BCF; (**c**) C0.050BCF; (**d**) C0.075BCF; (**e**) C0.100BCF; (**f**) C0.125BCF.

Figure 4 shows the FESEM images of CBCF materials with different Ce ion contents. As shown in Figure 4a, the pure BCF material displays the regular hexagonal platelet shape with the thickness of 500 nm and the diameter of 7 μm. The CBCF materials with Ce elements (Figure 4b–f) also exhibit a regular hexagonal shape, but with a small amount of unevenly distributed particles, which may be due to rapid crystal growth during the sintering process [34]. It is noticed that the size of the CBCF gradually decreases with the doping content of Ce, which may be due to the shift of grain boundaries and the inhibition of grain growth by the doping of rare earth ions [32,33]. All CBCF materials exhibit the same stacking and agglomeration, which may be due to the paramagnetic of W-type ferrite [34].

**Figure 5:**
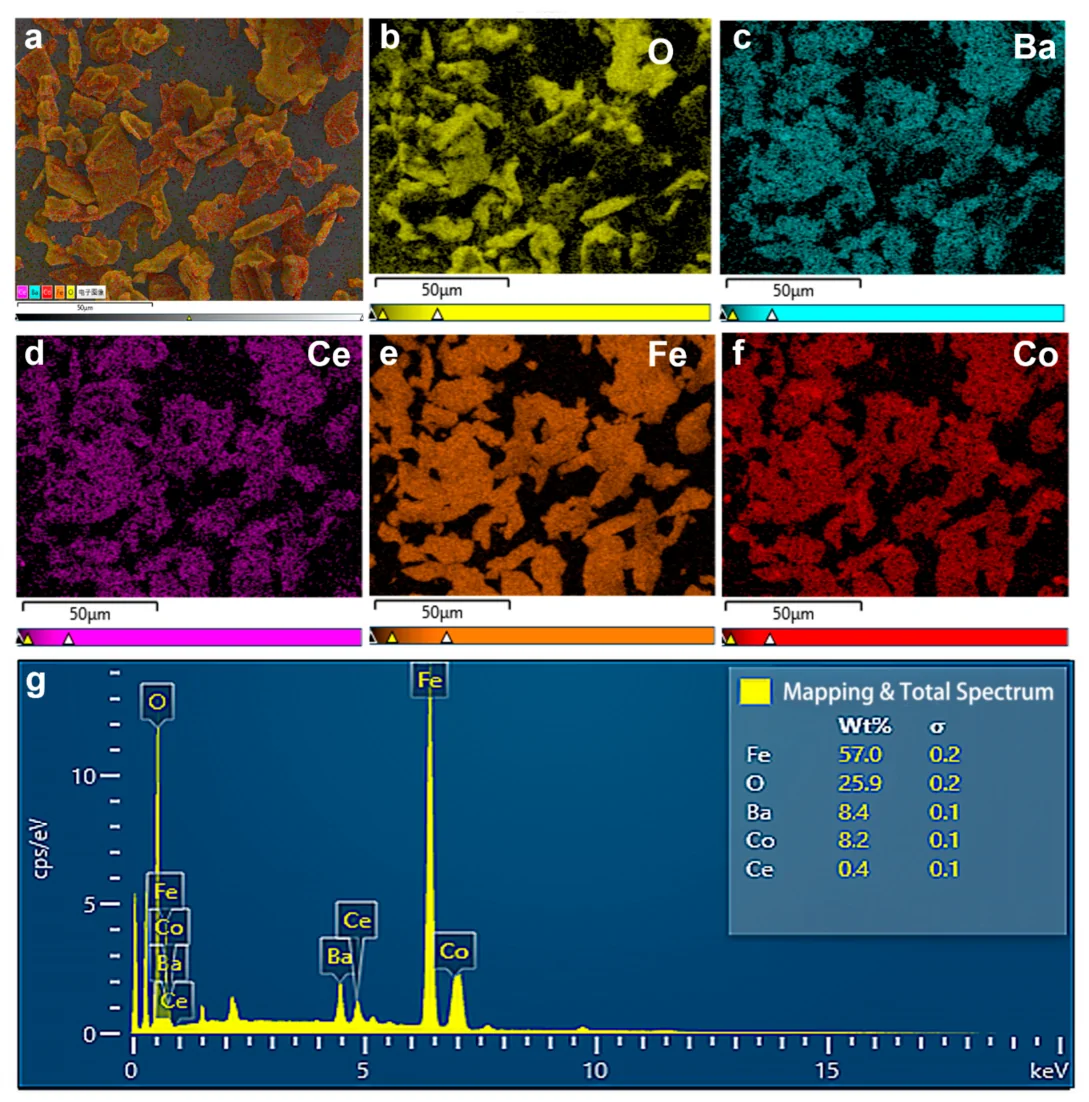
EDS of C_0.075_BCF. (**a**) Total elemental spectrogram; (**b**) O element; (**c**) Ba element; (**d**) Ce element; (**e**) Fe element; (**f**) Co element; (**g**) Energy dispersive spectrum. In the elemental mapping images (**b**–**f**), the different colors represent the distribution of the corresponding elements.

As shown in Figure 5, the elemental distribution of the C_0.075_BCF sample was determined by energy dispersive spectroscopy (EDS). Figure 5a–g shows that the elements of Fe, O, Co, Ba and Ce are evenly distributed in the CBCF material, and the proportions of Fe, O, Ba, Co and Ce were 57.0 wt%, 25.9 wt%, 8.4 wt%, 8.2 wt% and 0.4% wt%.

### 3.2. Microwave Absorption Performance

**Figure 6:**
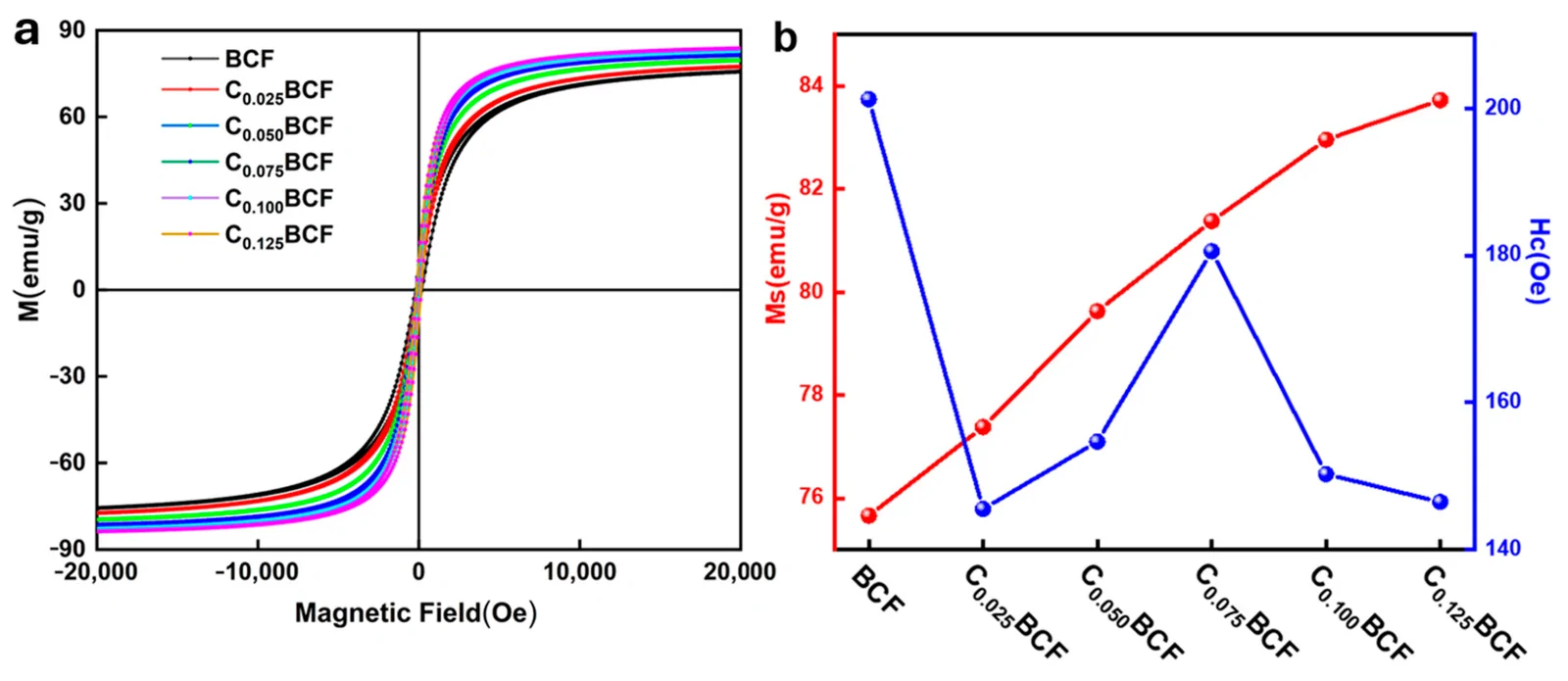
The static magnetic performance of CBCF samples: (**a**) the magnetic hysteresis loops; (**b**) Saturation magnetization (M_s_) and coercive field (H_c_).

The magnetic hysteresis loops of CBCF samples were measured at a range of −20 kOe to 20 kOe. The specific values of saturation magnetization (Ms), coercivity (Hc), and anisotropy field (H_a_) are shown in Table 2. The hysteresis loops were measured under a maximum applied field of ±20 kOe. To verify whether this field is sufficient to obtain reliable saturation magnetization, the magnetization curves were examined. All samples are nearly saturated above 15 kOe, with only a negligible slope remaining at 20 kOe. The extracted Ms values (75.66–83.73 emu/g) agree well with those reported for similar W-type hexaferrites in the literature (e.g., 78–80 emu/g [46]). Moreover, previous studies on W-type hexaferrites have successfully obtained reliable Ms values under a maximum field of 10 kOe [47], confirming that W-type ferrites can approach saturation at relatively low fields. Therefore, the data measured at ±20 kOe are considered sufficient to reflect the real magnetization of the samples. The trend of the hysteresis loop, Ms, and Hc of the materials with the increase in Ce ion contents is shown in Figure 6.

Based on Figure 6a and Table 2, CBCF materials exhibit high saturation magnetization and relatively low coercivity, which indicates that the Ce-doping W-type hexaferrite materials exhibit excellent soft magnetic properties [48,49]. This result is consistent with previous studies. Moreover, the shape of the hysteresis loop remains intact with increasing Ce doping content, indicating that the addition of dopants does not disrupt the magnetic behavior of the ferrite material. This suggests that CBCF materials can quickly reach saturation under an applied magnetic field and exhibit rapid magnetic relaxation when the external magnetic field is removed.

As shown in Figure 6b, the saturation magnetization of CBCF materials increases from 75.66 emu/g to 83.73 emu/g when Ce increases from 0 to 0.125, exhibiting excellent magnetic performance. This may result from the super-exchange interaction of Fe^3+^-O-Fe^3+^ [11]. In the structure of CBCF samples, Fe^3+^ mainly occupies octahedral (12*k*, 4*f*_2_ and 2*a*), tetrahedral (4f1), and trigonal bipyramidal (2b) sites, with a ratio of 10:1:2:4:1 [11]. In the case of magnetic ordering, the lattice spins at 12k, 2a, and 2b, where sites are parallel to the crystallographic C axis, while the spins at the 4f1 site are anti-parallel to the C axis. After Ce replaces Ba, the lattice parameter decreases and the corresponding Fe-O distance decreases, resulting in an enhanced super-exchange interaction of Fe^3+^-O-Fe^3+^ in the 12k and 2b sublattices.

The value of the coercive force of C_0.025_BCF decreases compared with BCF. The value of the coercive force increases by increasing the doped content from 0.025 to 0.075 and then decreases for x > 0.075 (Figure 6b). This indicates that cerium doping can significantly affect the coercive force of CBCF materials. According to ferromagnetic theory [50]:

(3) $$Hc=\frac{0.64K1}{\mathrm{Ms}}$$

In Equation (3): magnetic anisotropy constant, Ms: saturation magnetization.

At x = 0.025, the doping amount of cerium is relatively small, and it does not cause lattice distortion or an increase in magnetic anisotropy in the CBCF material. However, the increase in saturation magnetization leads to a rapid decrease in coercive force. As the doping amount increases, the lattice distortion caused by cerium enhances, leading to an increase in coercive force. When the doping amount reaches 0.100, the lattice distortion cannot continue to increase magnetic anisotropy, resulting in a subsequent decrease in coercive force [10,21].

The anisotropy field *H_a_* of CBCF materials were calculated by Equations (4) and (5), and the results shown in Table 2.

(4) $$M=M_{S}\left(1-\frac{A}{H}-\frac{B}{H^{2}}+\cdots \right)+\chi_{p}H$$

(5) $$B=\frac{1}{15}{Ha}^{2}$$

where A is the inhomogeneity parameter, $\chi_{p}$ is the high-field susceptibility, and B is the anisotropy parameter related to the magnetocrystalline anisotropy. When the high fields are higher than 1.5 kOe, M(H) and 1/H^2^ exhibit linear variation, and the parameters A and $\chi_{p}$ are negligible. As shown in Table 2, the H_a_ decreased with the increase in Ce^3+^ doped content.

**Figure 7:**
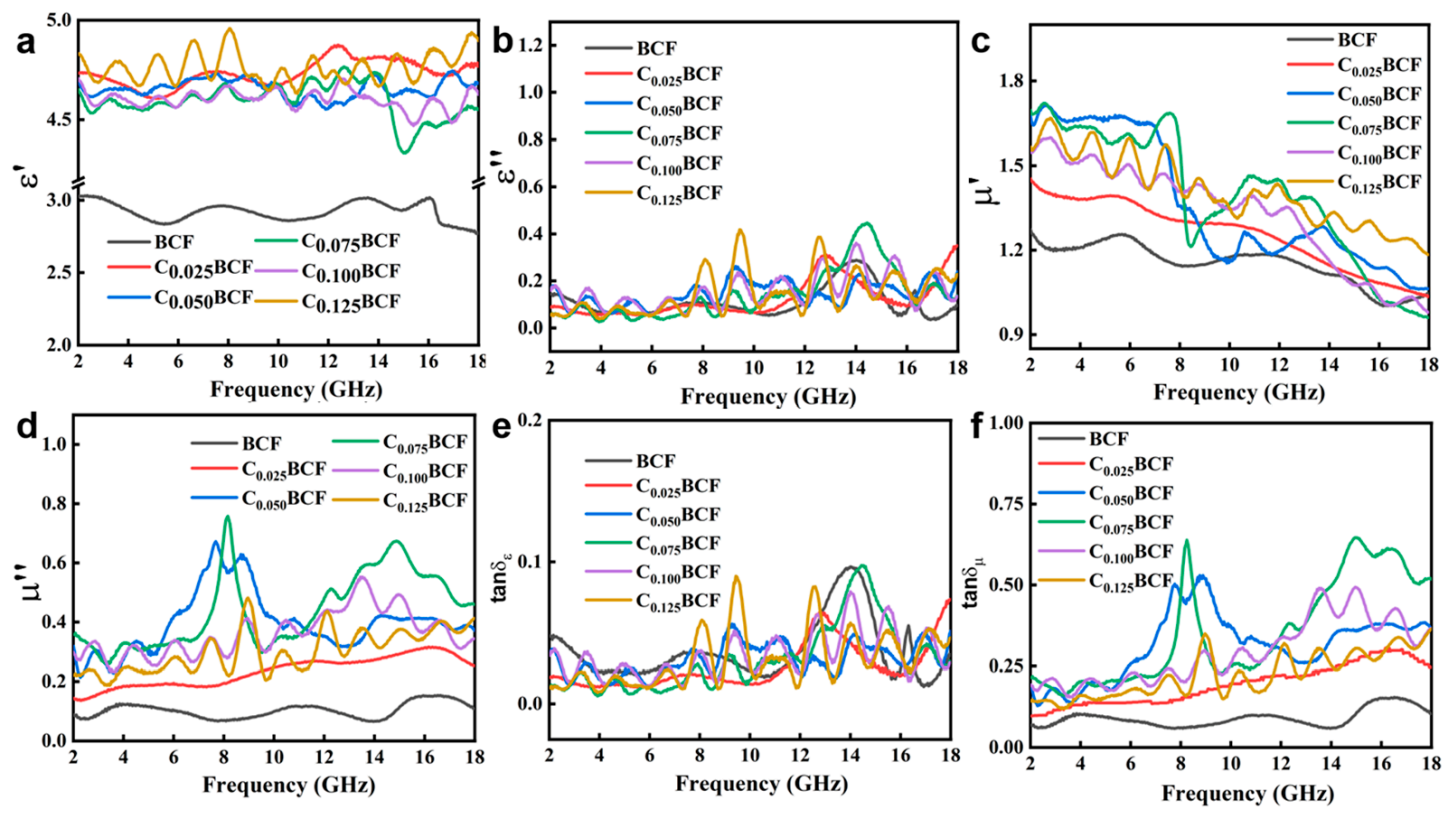
Frequency dependence of electromagnetic parameters for the prepared samples: (**a**) real permittivity (ε′), (**b**) imaginary permittivity (ε′′), (**c**) real permeability (μ′), (**d**) imaginary permeability (μ′′), (**e**) dielectric loss tangent (tanδ_ε_), and (**f**) magnetic loss tangent (tanδ_μ_).

Figure 7 shows the relative complex permittivity ε_r_ = ε′ − jε″ and relative complex permeability μ_r_ = μ′ − jμ″, which were measured at a frequency of 2–18 GHz for CBCF materials. Figure 7a exhibits that the real part of ε′ of BCF and CBCF materials fluctuates in a frequency range of 2–18 GHz. It can be observed that the real part ε′ of pure BCF material changes at 2.8–3, while the ε′ of CBCF materials with different doping levels fluctuates at 4.5–5. The results showed that the real part ε′ of CBCF is higher than that of BCF at a frequency of 2–18 GHz, which illustrates that the storage capacity of electromagnetic is enhanced [11,39]. From Figure 7b, with the increase in frequency, the imaginary part of ε″ for all materials also increases significantly. The pure BCF material only shows a peak at 12–16 GHz, which is due to the polarization effects of materials. On the contrary, the CBCF materials show multiple peaks in the range of 8–16 GHz. This is mainly attributed to charge polarization and defect polarization [39]. The difference in ionic radii and the heterovalent substitution between Ce^3+^ and Ba^2+^ leads to a distortion of the CBCF structure and more frequent electronic transitions between Fe^3+^ and O^2-^, enhancing the conductivity loss of CBCF and increasing the overall dielectric constant [10,39].

As shown in Figure 7c, compared with pure BCF ferrite, CBCF materials possess higher μ′ and μ″. The μ′ of original BCF and CBCF materials with different Ce^3+^ ion doping content decreases with increasing frequency and with many fluctuations. The μ′ of the original BCF material ranges from 0.9 to 1.2, while the μ′ of the doped CBCF materials varies between 1 and 1.8, which are all higher than that of the un-doped BCF material. This indicates that rare earth cerium doping can enhance the complex magnetic permeability of BCF. From Figure 7d, the imaginary part of μ″ of the pure BCF material changes slowly from 2 to 18 GHz without any obvious resonance peaks, which may be because the resonance frequency of the original BCF is higher than 18 GHz. In BCF, Co^2+^ ions occupy octahedral sites and form a planar anisotropy. The magnetic moments can rotate relatively easily within this plane, resulting in a low coercive field and soft magnetic behavior. However, pulling the magnetic moments out of this plane requires a large amount of energy, which is determined by the magnetocrystalline anisotropy field (H_a_). The natural resonance frequency depends on H_a_, not on the coercive field. Therefore, a low coercive field does not imply a low resonance frequency, and the natural resonance frequency of the undoped sample remains above 18 GHz. In contrast, CBCF materials with different doping levels show distinct resonance peaks in the range of 2 to 18 GHz, which appear at 6 to 10 GHz and 12 to 16 GHz, indicating that rare earth cerium can effectively regulate the resonance frequency of BCF materials [11,42]. Based on ferromagnetic resonance theory, the natural resonance frequency is directly related to the magnetocrystalline anisotropy field. For the undoped BCF sample, although the planar magnetic anisotropy gives rise to soft magnetic behavior with low coercivity, the anisotropy field remains relatively large, placing its natural resonance frequency above 18 GHz. This explains why no distinct resonance peak is observed in the μ″ spectrum within 2–18 GHz. With increasing Ce content, the substitution of Ba^2+^ by Ce^3+^ induces lattice contraction and partial reduction of Fe^3+^ to Fe^2+^, which weakens the super-exchange interaction and modifies the local coordination environment of Co^2+^ at octahedral sites. Consequently, the anisotropy field *H_a_* decreases from 8.20 kOe (x = 0) to 5.65 kOe (x = 0.125), as listed in Table 2. This reduction shifts the natural resonance frequency into the 2–18 GHz range, manifesting as multiple resonance peaks in the μ″ curves at 6–10 GHz and 12–16 GHz. These results indicate that Ce doping effectively tunes the natural resonance frequency by lowering the magnetocrystalline anisotropy field, thereby contributing to the enhanced microwave absorption performance. The ferromagnetic resonance frequency was affected by *H_a_* as shown in Equation (6):

(6) $$f_{\mathrm{FMR}}=\frac{\gamma \mu_{0}}{2\pi }H_{a}$$

where *γ* is the gyromagnetic ratio and $\mu_{0}$ represents the permeability of vacuum. The peaks in the materials were attributed to the decrease in natural resonance frequency, which follows the changes in *H_a_.* After Ce doping, the coercive field and saturation magnetization show only minor changes, whereas the natural resonance frequency shifts significantly from above 18 GHz to the X-band. This is because the resonance frequency is governed by the magnetocrystalline anisotropy field H_a_. The substitution of Ce^3+^ induces lattice contraction and partial reduction of Fe^3+^ to Fe^2+^, thereby modifying the local coordination environment of Co^2+^ at octahedral sites and weakening the Co^2+^-Fe^3+^ super-exchange interaction. As a result, H_a_ decreases from 8.20 kOe (x = 0) to 5.65 kOe (x = 0.125) (Table 2), leading to a pronounced decrease in the natural resonance frequency. In addition, H_c_ is also influenced by domain wall pinning, grain size, and defects, which are not sensitive to Ce doping, while M_s_ is mainly determined by the Fe^3+^-O-Fe^3+^ super-exchange and also changes only slightly. Additionally, the natural resonance remains within a wide range.

To determine the dominant loss type of CBCF materials, the dielectric loss and magnetic loss of BCF and CBCF materials were calculated by the formulas tanδ_ε_ = ε″/ε′ and tanδ_μ_ = μ″/μ′, as shown in Figure 7e,f. From Figure 7e, the original BCF material exhibits a distinct loss peak at 12–16 GHz and the doped CBCF materials exhibit a significant increase in fluctuations, with multiple loss peaks appearing in the range of 8–16 GHz. This may be attributed to the interface polarization and defect polarization caused by Ce doping [39]. The tanδ_ε_ of CBCF materials with different Ce ion contents is stronger than that of pure BCF, but the increase is small. The tangent dielectric loss of all materials ranges from 0 to 0.1, indicating that the dielectric loss of BCF and CBCF materials is weak. As shown in Figure 7f, the original BCF material exhibits flat fluctuation in the range of 2–18 GHz. The CBCF materials exhibit distinct loss peaks at 6–10 GHz and 12–18 GHz, and the loss values were higher than those of the pure BCF material. This may be attributed to the shift of natural resonance peaks caused by the increase in Ce^3+^ ion concentration. Compared with Figure 7e,f, the tangent dielectric loss of the doped CBCF materials was lower than the magnetic loss, indicating that the main loss mechanism in CBCF samples is magnetic loss. Additionally, the wider loss peak can improve the effective absorption bandwidth.

The reflection loss (R*_L_*), which was used to indicated the microwave absorbing properties, was calculated by the equation [51]:

*R_L_* = 20log|(Z_in_ − Z_0_)/(Z_in_ + Z_0_)| (7)

where Z_in_ is the normalized input impedance of the absorber and Z_0_ is the impedance of free space. Z_in_ can be obtained from the following equation:

(8) $$Z_{\mathrm{in}}=Z_{0}{\left(\mu_{r}/\varepsilon_{r}\right)}^{1/2}\tan h[j\left(2\pi fd/c\right)\left(\mu_{r}\varepsilon_{r}{)}^{1/2}\right]$$

where ε_r_ and μ_r_ are the relative complex permittivity and permeability, respectively; c is the velocity of electromagnetic waves in free space; f is the frequency of microwave; and d is the thickness of the absorbers.

**Figure 8:**
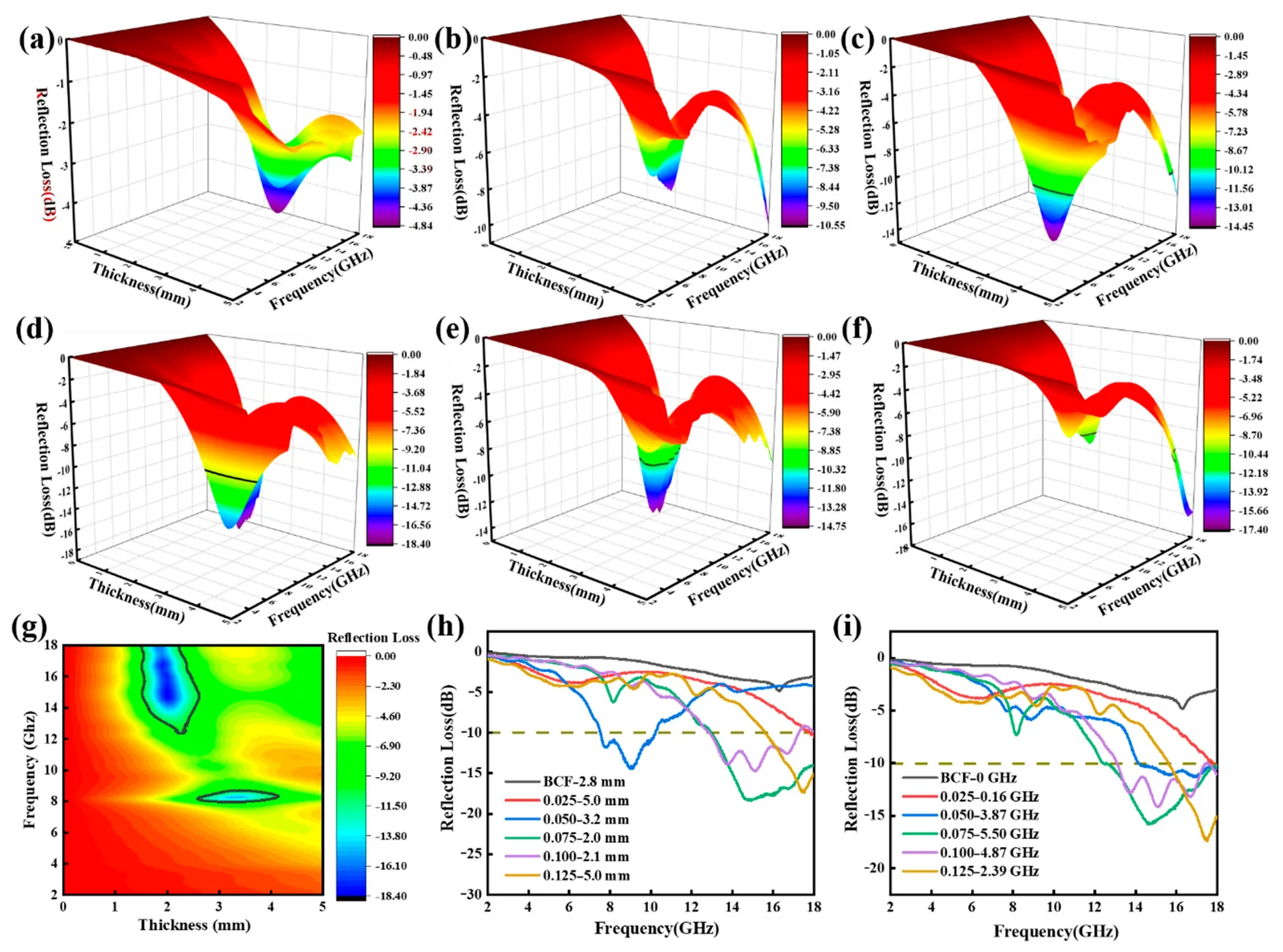
Microwave absorption performance of the samples: 3D reflection loss (RL) curves of (**a**) BCF, (**b**) C_0.025_BCF, (**c**) C_0.05_BCF, (**d**) C_0.075_BCF, (**e**) C_0.1_BCF, and (**f**) C_0.125_BCF; (**g**) 2D contour plot of reflection loss for the C_0.075_BCF sample; (**h**) the minimum reflection loss (RLmin) and (**i**) the maximum effective absorption bandwidth (EABmax) as a function of frequency for different samples, The horizontal dashed line indicates the -10 dB threshold.

The 3D reflection loss of CBCF materials is presented in Figure 8. It can be observed that the minimum reflection loss (RL_min_) of BCF, C_0.025_BCF, C_0.050_BCF, C_0.075_BCF, C_0.100_BCF, and C_0.125_BCF, is −4.84 dB, −10.55 dB, −14.45 dB, −18.40 dB, −14.75 dB, and −17.40 dB, respectively. The RL_min_ of the doped CBCF samples is better than that of BCF; this may be attributed to the stronger imaginary parts of complex permeability in CBCF materials. For the samples with different Ce doping levels, the RL_min_ values correspond to frequencies of 16.28 GHz, 18.00 GHz, 9.00 GHz, 14.92 GHz, 15.12 GHz, and 17.52 GHz, respectively. Meanwhile, the multiple resonance peaks observed in Figure 8 originate from two distinct mechanisms. First, the substitution of Ce^3+^ for Ba^2+^ induces partial reduction of Fe^3+^ to Fe^2+^, generating new Landé g-factors and multiple natural resonance modes. These intrinsic resonances appear as distinct peaks in the μ″ spectrum at 6–10 GHz and 12–16 GHz (Figure 7) and do not shift with thickness. Second, thickness-induced λ/4 interference causes some absorption peaks to shift to lower frequencies as the thickness increases, following the quarter-wavelength matching condition [52]. The overlap of these two types of peaks broadens the effective absorption bandwidth and enhances the overall absorption performance. With an increase in rare earth cerium doping content, the frequency of CBCF materials shifts towards lower frequencies first and then towards higher frequencies. This phenomenon was attributed to the shift of natural resonance frequency, which was affected by Ce^3+^ ions’ substituted concentration.

As shown in Figure 8, the effective absorption bandwidth (EAB) of pure BCF is almost zero, while the EAB of C_x_B_1-x_CF is 0.16 GHz, 3.87 GHz, 5.52 GHz, 4.87 GHz, and 2.39 GHz, respectively. Additionally, the microwave-absorbing properties of CBCF samples were concentrated at 12–18 GHz, which was consistent with the imaginary part of μ″. The improvement in the microwave absorbing performance of CBCF was attributed to the reduction in natural resonance frequency and the maintain magnetic loss, which can be seen in Figure 7f.

**Figure 9:**
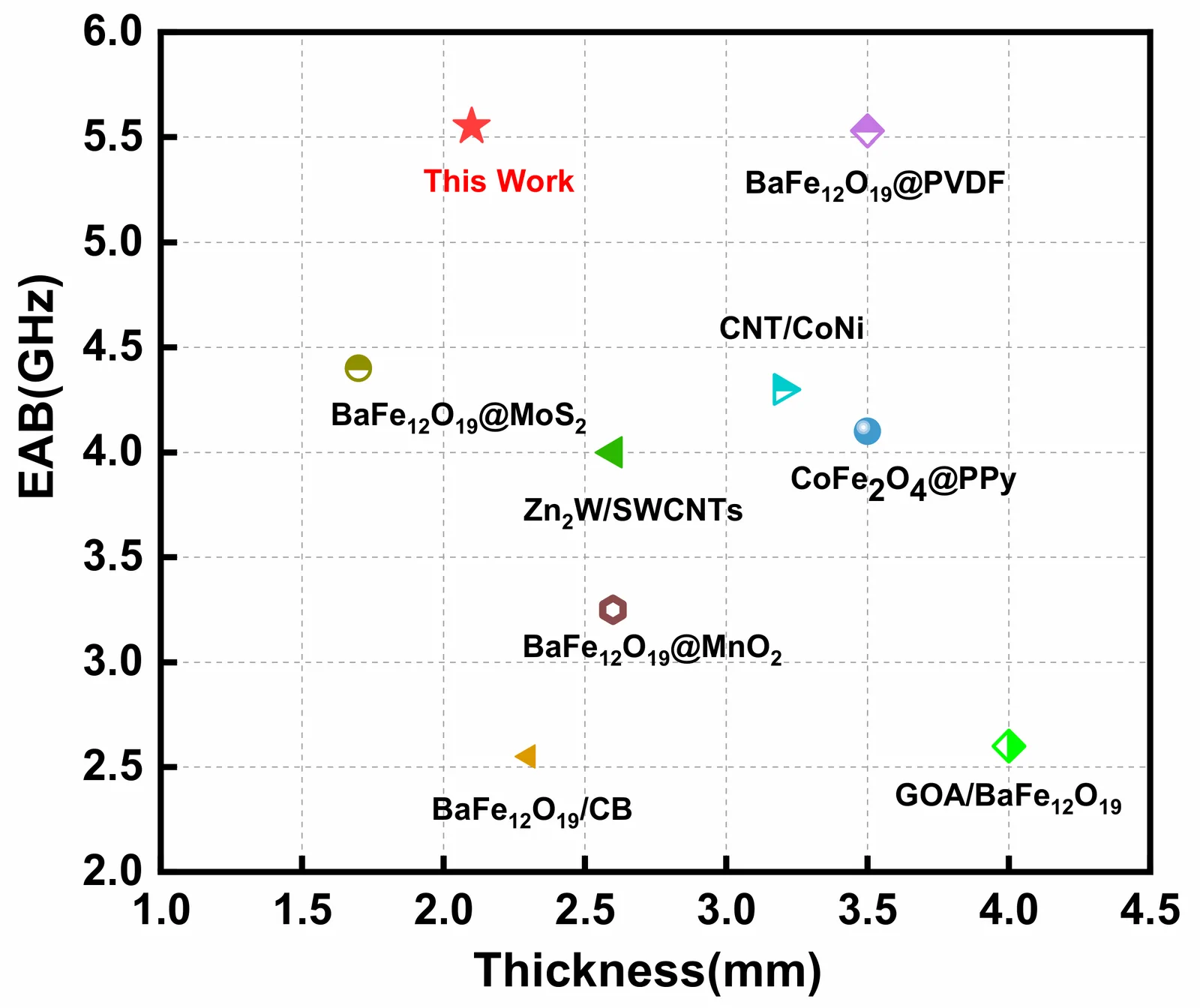
The comparison of EABmax performance with reported ferrite-based composite.

The EAB performance of prepared C_0.075_BCF samples was compared with the reported ferrite-based composite, as shown in Figure 9. The rare earth doping hexaferrite prepared in this article displays a wider effective absorption bandwidth compared with a ferrite-based composite because of the higher EAB at low thickness.

**Figure 10:**
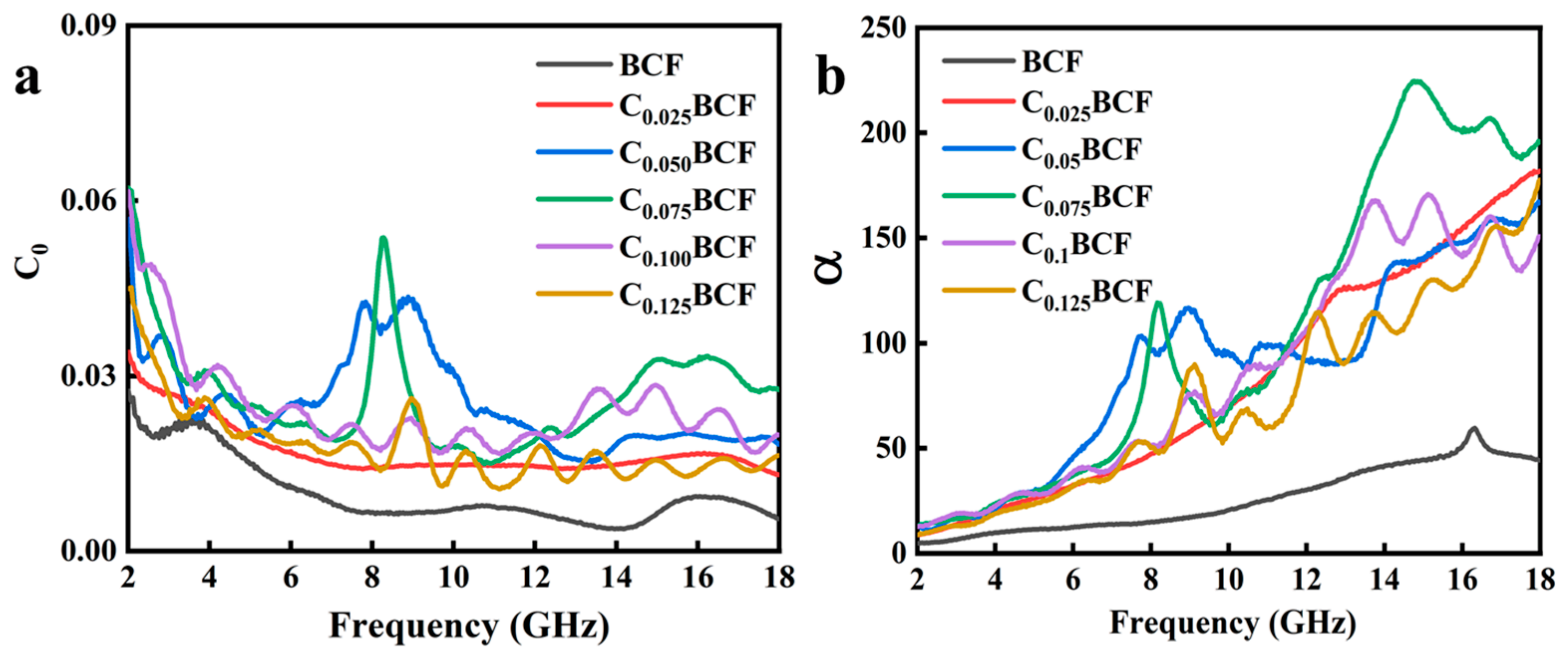
Frequency dependence of (**a**) the eddy current coefficient (C_0_) and (**b**) the attenuation constant (α) for the prepared samples.

As shown in Figure 10a, the eddy current coefficient (C_0_) was determined by Equation (9) [50]:

(9) $$C_{0}=\mu^{\prime \prime }{\left(\mu^{\prime }\right)}^{-2}f^{-1}=2\pi \mu_{0}\sigma d^{2}/3$$

where μ_0_ is the initial permeability and σ is the electrical conductivity. The microwave absorption mechanization of magnetic materials includes domain wall resonance, hysteresis, the eddy current effect and natural resonance [50]. Generally, the eddy current effect and natural resonance mainly dominate loss in the frequency of GHz. The C_0_ of original BCF material decreases gradually with increasing frequency, and any significant peak no longer appears, indicating that the BCF material has only eddy current loss in the range of 2–18 GHz. This may be because the natural resonance frequency of BCF material is higher than 18 GHz [53]. With the addition of Ce elements, CBCF materials exhibit distinct loss peaks in the ranges of 6–10 GHz and 14–18 GHz, with values greater than those of the BCF material. This indicates that CBCF materials, in addition to eddy current loss, also have natural resonance loss, thanks to the rare earth ions adjusting the natural resonance frequency of BCF [54].

As seen in Figure 10b, the attenuation constant (α) was determined by Equation (10) [55]:

(10) $$\alpha =\frac{\sqrt{2}\mathrm{\pi f}}{C}\times \sqrt{\left(\mu^{\prime \prime }\varepsilon \prime \prime -\mu^{\prime }\varepsilon^{\prime }\right)+\sqrt{{\left(\mu^{\prime \prime }\varepsilon \prime \prime -\mu^{\prime }\varepsilon^{\prime }\right)}^{2}+{\left(\mu^{\prime }\varepsilon \prime \prime +\mu^{\prime \prime }\varepsilon^{\prime }\right)}^{2}}}$$

The BCF presents a lower value of α without obvious peaks. CBCF materials with different doping levels, which exhibit a gradual increase in the loss coefficient with frequency and have peaks at 6–10 GHz and 12–18 GHz, which shows that the CBCF materials have excellent microwave performance. Additionally, the C_0.075_BCF materials reach the maximum value and maintain a wide range, which is the reason for the thin thickness (2.1 mm) and larger EAB (5.52 GHz) [50,54].

**Figure 11:**
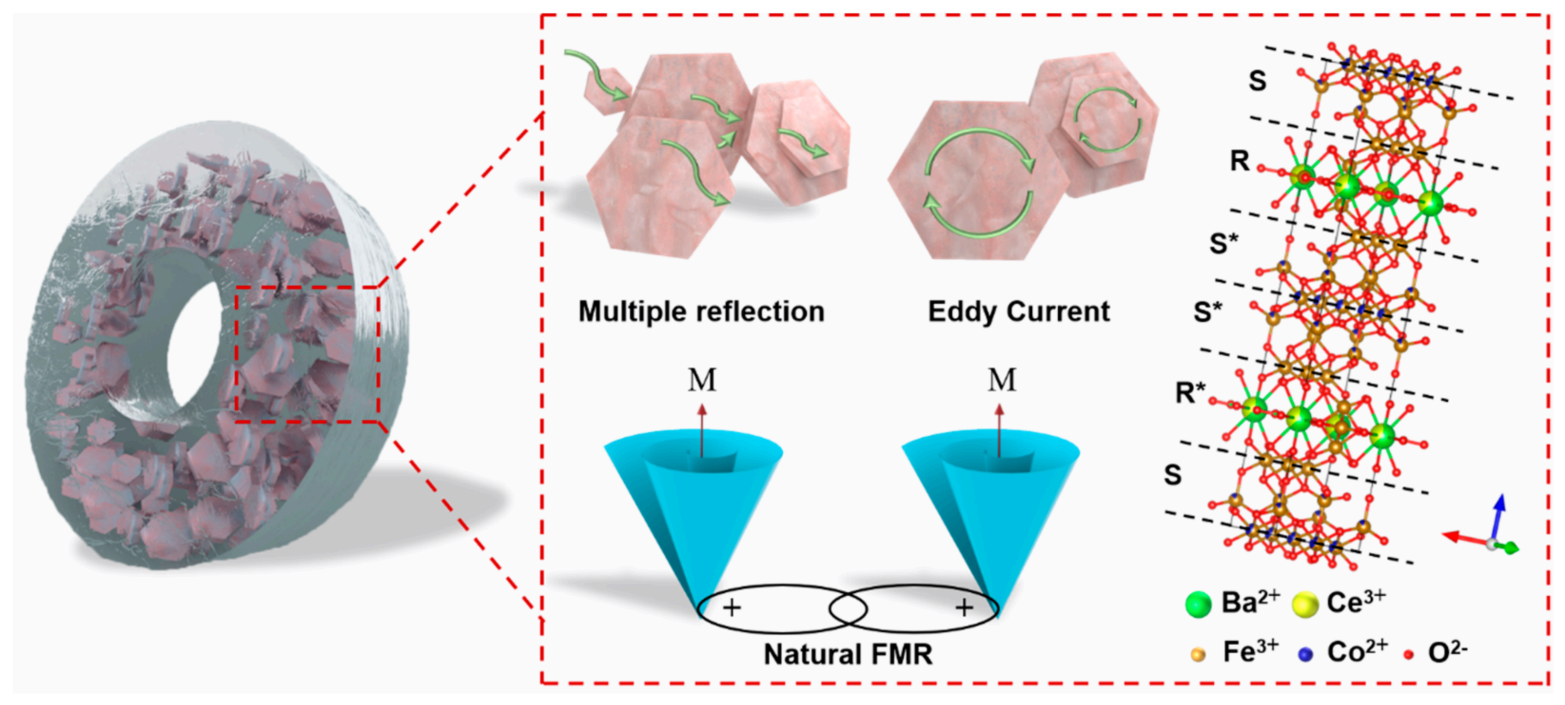
The microwave absorption mechanism of Ce^3+^-doped hexaferrite. The green arrows indicate multiple reflections and eddy currents. The blue cones represent the natural ferromagnetic resonance (FMR). The crystal structure illustrates the atomic arrangement and the heterovalent substitution of Ba^2+^ by Ce^3+^. The asterisk (*) indicates the specific crystallographic site (e.g., the octahedral site) occupied by the metal ions.

The broadband microwave absorption mechanism of the Ce^3+^-doped W-type hexaferrite is illustrated in Figure 11. The natural resonant frequency of W-type hexaferrite was adjusted by the rare earth doping, which can be confirmed in Figure 5 and Figure 6. Moreover, the analysis of absorption performance proved that the absorption frequency was determined by the natural resonant loss, which can enhance the magnetic loss at a wider frequency range. Finally, C_0.075_BCF materials showed excellent absorption performance, with an RL of −18.40 dB at 14.92 GHz and an effective absorption bandwidth of 5.52 GHz.

## 4. Conclusions

In this work, Ce-doped W-type hexagonal ferrites (C_x_BCF, x = 0, 0.025, 0.050, 0.075, 0.100, 0.125) were successfully synthesized via the sol–gel method. The results demonstrate that Ce doping does not disrupt the formation of the W-type phase, and the materials maintain a typical hexagonal sheet structure. As the Ce doping content increases, the saturation magnetization gradually increases, reaching a maximum value of 83.73 emu/g at x = 0.125. This enhancement is primarily attributed to the improved crystallinity and the strengthened Fe^3+^-O-Fe^3+^ super-exchange interaction. Furthermore, the introduction of Ce effectively modulates the natural resonance frequency and electromagnetic parameters of the W-type ferrite, thereby optimizing the impedance matching. The frequency corresponding to the maximum reflection loss can be tuned by varying the Ce content. Notably, the C_0_._075_BCF sample exhibited effective broadband absorption, achieving a minimum reflection loss of −18.40 dB at 14.92 GHz and an effective absorption bandwidth of 5.52 GHz. This study provides a promising strategy for designing frequency-tunable broadband microwave absorbers. Additionally, exploring other dopants with strong electric dipole polarization (such as Bi) may serve as a complementary approach to further tune the dielectric loss in future work.

## Figures and Tables

**Table 1:** Calculated lattice parameters of CBCF and obtained fitting parameters.

Content	a	c	c/a	V_cell_	χ2
0	5.9104	33.0158	5.586	998.788	2.3%
0.025	5.9045	32.9645	5.583	995.246	2.1%
0.05	5.9035	32.9187	5.576	993.527	3.2%
0.075	5.8977	32.8764	5.574	990.302	2.7%
0.1	5.8864	32.8645	5.583	986.153	9.4%

**Table 2:** Saturation magnetization (M_S_), coercive field (H_C_), and anisotropy field (H_a_) values of the Ce^3+^ substituted W-type hexaferrites.

Content	Ms (emu/g)	Hc (Oe)	Ha (kOe)
0	75.66	201.23	8.20
0.025	77.38	145.52	7.76
0.050	79.63	154.68	6.77
0.075	81.38	180.56	6.22
0.100	82.96	150.26	5.85
0.125	83.73	146.47	5.65

## Data Availability

The original contributions presented in this study are included in the article. Further inquiries can be directed to the corresponding author.
